# Pharmacodynamic Activity of the Novel Neurokinin-3 Receptor Antagonist SJX-653 in Healthy Men

**DOI:** 10.1210/clinem/dgaa657

**Published:** 2020-09-18

**Authors:** Richard A Anderson, Jennifer Cormier, Ruth Thieroff-Ekerdt, Malcolm Boyce, Frans van den Berg, Daniel Grau, David Turnquist, Deya Corzo, Philip Graham

**Affiliations:** 1 MRC Centre for Reproductive Health, University of Edinburgh, Edinburgh, UK; 2 Sojournix, Inc., Waltham, Massachusetts; 3 Hammersmith Medicines Research, London, UK

**Keywords:** neurokinin, NK3 antagonist, KNDy neuron, LH, testosterone, pharmacodynamics

## Abstract

**Context:**

SJX-653 is a novel neurokinin 3 receptor (NK3R) antagonist. The NK3 pathway is a central regulator of gonadotropin releasing hormone (GnRH) secretion and has also been implicated in the generation of hot flashes. Therefore, decreases of luteinizing hormone (LH) and testosterone in men serve as sensitive pharmacodynamic (PD) markers of central NK3 antagonism.

**Objective:**

To characterize the safety, tolerability, pharmacokinetics, and pharmacodynamic activity of SJX-653 in healthy men.

**Design:**

A randomized, placebo-controlled, double-blind, single ascending dose study.

**Setting:**

Phase 1 unit.

**Patients or Other Participants:**

Seven cohorts of 6 healthy men 18–45 years of age (4:2 randomization to SJX-653/placebo per cohort).

**Intervention(s):**

Single oral doses of 0.5–90 mg SJX-653.

**Main Outcome Measure(s):**

Safety assessments and serial pharmacokinetic (PK)/PD measurements.

**Results:**

SJX-653 was well tolerated at all dose levels. C_max_ and AUC_0-24_ increased in a dose-proportional manner. The terminal elimination half-life ranged between 9.8 and 12.5 hours independent of dose. A statistically significant, dose-dependent, reversible reduction of LH and testosterone was observed with near maximal effect after 15 mg and little to no effect at 4.5 mg. Maximal LH reduction was 70 ± 7% (mean ± sd) at 6 hours after 30 mg SJX-653 versus 10 ± 43% for placebo (*P* = 0.0006); maximal T reduction was of 68 ± 5% at 8 hours after 60 mg SJX-653 versus 18 ± 11% for placebo (*P* < 0.0001). The plasma IC_50_ for LH reduction was 33 ng/mL.

**Conclusions:**

These data demonstrate clinical proof-of-mechanism for SJX-653 as a potent centrally-acting NK3R antagonist.

Preclinical data indicate that SJX-653 is a centrally active, competitive antagonist of the human neurokinin 3 (NK3) receptor. It is based on a proprietary isoquinolinone scaffold originally developed by H. Lundbeck A/S (1, 2), which is chemically distinct from other NK3 antagonists currently or previously in clinical development and has >1000-fold selectivity versus NK1 and >100-fold versus NK2 (Sojournix, data on file).

Neurokinin B/neurokinin 3 receptor (NKB/NK3R) signaling is now recognized to have a key role in the regulation of gonadotropin releasing hormone (GnRH) secretion in humans and thus acts as a major regulator of the hypothalamo-pituitary-gonadal (HPG) axis (3–5). Individuals with loss-of-function mutations in the genes encoding NKB and NK3R (*TAK3* and *TAK3R,* respectively) fail to undergo puberty due to hypogonadotropic hypogonadism (4). Suppression of GnRH secretion or action is the basis for a wide range of medical treatments, including that of uterine fibroids, endometriosis, and prostate cancer, as well as underpinning approaches to ovarian stimulation in assisted reproduction and indeed hormonal contraception. Novel therapies targeting GnRH secretion may therefore have a wide application in sex hormone–dependent and other conditions. The essential role of the NKB/NK3R pathway in regulating GnRH secretion in men and women has been confirmed through studies with NK3R antagonists. These have shown efficacy in suppressing luteinizing hormone (LH) and testosterone secretion in men (6, 7), and gonadotropin secretion, follicle growth, and ovulation in premenopausal women (6, 8–10). Suppression of LH and testosterone secretion has also been demonstrated in women with polycystic ovary syndrome (PCOS) (11). These findings show proof of concept for the potential efficacy of NK3R antagonists in hormone-dependent conditions, and while confirmation of both efficacy and comparison with existing therapies are awaited, their apparent specificity and potential for variable rather than complete suppression of the HPG axis in both sexes may have therapeutic advantages. NKB can be expressed in the same neurons in the hypothalamus as kisspeptin (with dynorphin also expressed in some, giving rise to the term KNDy neurons) (5), with NKB signaling being functionally upstream of kisspeptin (10, 12, 13). As a result, reductions of LH and testosterone in men are now established pharmacodynamic (PD) markers of central NK3R antagonism in men, and doses of NK3R antagonists that reduce levels of these markers in men may inform doses across a range of indications of conditions in which NK3R signaling is implicated.

NKB signaling through the NK3 receptor (NK3R) also has a key role in the generation of menopausal vasomotor symptoms (VMS), commonly called “hot flashes.” Specifically, excessive NKB/NK3 signaling in the preoptic area of the brain leads to dysfunctional thermoregulation in conditions of low estrogen, such as the postmenopausal state (14, 15), and elicits an excessive heat dissipation response, experienced symptomatically as VMS. Consistent with this role of NKB/NK3R signaling in the generation of VMS, a growing body of evidence shows that NK3 receptor antagonists cause rapid and profound suppression of menopausal VMS in women (9, 16–18), with this approach having the potential to offer a highly effective nonhormonal therapy for that condition. In addition, a genome-wide association study (19) of almost 18 000 postmenopausal women linked self-reported VMS to 14 single-nucleotide polymorphisms all located on chromosome 4 in the locus encoding the NK3 receptor. The effectiveness of 2 NK3R antagonists at suppressing VMS occurs at similar doses to those previously shown to be effective at suppressing LH secretion in men (6, 7).

The objectives of this study were to evaluate the safety, tolerability, pharmacokinetics (PK), and PD of single ascending oral doses of a novel and selective NK3R antagonist, SJX-653, in healthy adult men in order to predict effective doses for application in VMS and also potentially for application in a range of sex hormone–dependent conditions.

## Materials and Methods

### Subjects and protocol

This study was a randomized, placebo-controlled, double-blind, single ascending dose study of orally administered SJX-653. Eligible subjects were healthy men aged 18–45 years, nonsmokers, with body mass index between 18 and 32 kg/m^2^, with a normal physical examination, normal hematology, biochemistry and urinalysis, and normal electrocardiogram. The lower cutoff for total testosterone (hereafter “testosterone”) was 8.5 nmol/L. The study was conducted in compliance with the International Committee on Harmonisation-Good Clinical Practice guidance, and all subjects provided informed written consent. Potential subjects were assessed for eligibility at an initial screening visit; eligible subjects were admitted to the clinical site (Hammersmith Medicines Research) on Day 1 and were randomized on Day 1. Forty-two subjects were randomly assigned 4:2 to receive SJX-653 or matching placebo in a fasted state to 7 increasing dose cohorts (0.5, 1.5, 4.5, 15, 30, 60, and 90 mg). The initial dose cohort had a sentinel group of 2 subjects who received either SJX-653 or placebo (1:1 randomization) before dosing the remaining subjects. Eligible subjects were confined to the clinical site for the duration of the in-house period, administered a single dose of SJX-653 on Day 1, discharged from the clinic on the morning of Day 3, and returned to the clinical site on Day 8 (±2 days) for end-of-study safety assessments (including physical examination, hematology, biochemistry, urinalysis, and electrocardiogram). Cohorts 5, 6, and 7 (30, 60, and 90 mg doses) also returned to the clinic on Day 4 for PK plasma collection, 72 hours after drug administration.

A Safety Review Committee was established to determine and approve the dose levels administered during dose escalation, following a blinded review of the safety, tolerability, and anonymized PK and PD data for each previous dose cohort. The lowest dose was based on a review of preclinical toxicity data with a wide margin for safety. The dose was increased until a maximal PD response appeared to have been achieved, and plasma levels of SJX-653 suggested a high level of NK3 antagonism.

### Pharmacokinetic and pharmacodynamic assessments

The PK and PD (LH, FSH, and testosterone) of SJX-653 were assessed by the collection of serial blood samples predose (-1.5, -1, -0.5, 0 hours) and at 0.5, 1, 1.5, 2, 2.5, 3, 4, 6, 8, 12, 16, 24, 36, and 48 hours postdose, with an additional sample at 72 hours for the 3 highest dose cohorts. PK analyses were performed using liquid chromatography/tandem mass spectrometry (LC/MS/MS). The limit of quantitation for the PK assay was 0.200 ng/mL.

Levels of serum FSH and LH were measured using UniCel DxI 800 Immunoassay analyzer using Beckmann-Coulter Access sequential 2-step immunoenzymatic assay system. Testosterone was measured using the UniCel DxI 800 immunoassay analyzer with a competitive binding immunoenzymatic assay using Beckmann-Coulter reagents. The limit of quantitation was 0.2 IU/L for FSH, 0.2 IU/L for LH, and 0.4 nmol/L for testosterone. The lowest QC samples and corresponding within-run CV% were: FSH 6.5 IU/L (4.7%), LH 4.29 IU/L (4.3%), and T 2.7 nmol/L (9.6%).

### Statistical analysis

All descriptive statistical analyses were performed using Statistical Analysis Software Version 9.4. Phoenix WinNonlin (Version 8.1) to estimate all PK parameters using the noncompartmental analysis tool following the Linear Trapezoidal Linear Interpolation calculation method. For each PD marker at each dose, postdose concentration values were converted to ratios versus the predose value (mean of -1.5 hour, -1.0 hour, -0.5 hour, and 0 hours). The maximum 1 − ratio from baseline, and the time area under baseline were statistically compared for each PD marker in each SJX-653 dose cohort versus placebo, using the nonparametric Wilcoxon rank-sum test. For each dose cohort, the least square means concentration of LH, FSH, and testosterone were compared with placebo at each collection time point using an analysis of covariance (ANCOVA) model, with the baseline concentration as covariate. No statistical techniques were used to calculate the sample size: the numbers of subjects were chosen based on feasibility and if they were considered sufficient to meet the study objectives.

### Regulatory and ethics committee approval

The Medicine and Healthcare products Regulatory Agency (EudraCT 2018-000376-16) and the Northern Ireland Research Ethics Committee B (118/NI/0075) approved the study.

## Results

In total, 42 subjects were enrolled into 7 dose cohorts (0.5, 1.5, 4.5, 15, 30, 60, and 90 mg), 28 subjects received SJX-653, and 14 received placebo. There were no withdrawals. Subject demographics are shown in Table 1.

**Table 1.** Subject demographics and baseline characteristics

	Placebo (N = 14)	SJX-653 (N = 28)
Age (years)		
Mean (SD)	30.9 (8.03)	33.1 (8.20)
Min, max	21, 45	19, 45
Height (cm)		
Mean (SD)	177.14 (7.33)	177.82 (6.83)
Min, max	162.0, 187.0	162.0, 191.0
Weight (kg)		
Mean (SD)	78.81 (8.96)	79.01 (12.44)
Min, max	63.1, 99.7	55.9, 105.9
BMI (kg/m^2^)		
Mean (SD)	25.09 (2.20)	24.93 (3.12)
Min, max	20.8, 29.4	19.3, 29.7
Race		
Asian	0	3 (10.7)
Black/African American	5 (35.7)	6 (21.4)
White	8 (57.1)	18 (64.3)
Other	1 (7.1)	1 (3.6)

Abbreviations: BMI, body mass index; max, maximum; min, minimum; SD, standard deviation.

### Safety

SJX-653 was safe and well tolerated. Overall, 9/28 (32%) subjects treated with SJX-653 experienced at least 1 treatment-emergent adverse event (TEAE): 1 subject each in the 0.5-, 1.5-, 4.5-, 30-, and 60-mg cohorts, and 2 subjects each in the 15- and 90-mg cohorts. In the placebo group, 3/14 (21%) subjects experienced at least 1 TEAE. The majority of TEAEs were mild, none were severe. No serious adverse events (SAEs) were reported and no subjects discontinued the study due to a TEAE. A listing of adverse events by preferred term is given in Table 2. There were no clinically meaningful changes in vital signs, laboratory parameters, or electrocardiogram (ECG) parameters.

**Table 2.** Treatment-emergent adverse events by preferred term

Preferred Term	Placebo (n = 14)	SJX-653 dose							
		0.5 mg (n = 4)	1.5 mg (n = 4)	4.5 mg (n = 4)	15 mg (n = 4)	30 mg (n = 4)	60 mg (n = 4)	90 mg (n = 4)	All SJX-653
At least 1 TEAE	3 (21.4)	1 (25.0)	1 (25.0)	1 (25.0)	2 (50.0)	1 (25.0)	1 (25.0)	2 (50.0)	9 (32.1)
Headache	1 (7.1)	0	0	0	0	1 (25.0)	0	2 (50.0)	3 (10.7)
Somnolence	0	0	0	0	0	0	1 (25.0)	0	1 (3.6)
Abdominal pain	0	0	0	0	1 (25.0)	0	0	0	1 (3.6)
Diarrhea	0	0	0	0	1 (25.0)	0	0	0	1 (3.6)
Nausea	0	0	0	1 (25.0)	0	0	0	0	1 (3.6)
Back pain	0	0	1 (25.0)	0	0	0	0	0	1 (3.6)
Musculoskeletal pain	0	0	0	0	1 (25.0)	0	0	0	1 (3.6)
Catheter site pain	0	1 (25.0)	0	0	0	0	0	0	1 (3.6)
Nasopharyngitis	1 (7.1)	0	0	0	0	0	0	0	0
Night sweats	1 (7.1)	0	0	0	0	0	0	0	0

Data are n (%). AEs were summarized by subject incidence rates; therefore, a subject contributed only once to the count for a given AE.

Abbreviations: AE, adverse event; TEAE, treatment-emergent adverse event.

### Pharmacokinetics

**Figure 1.**
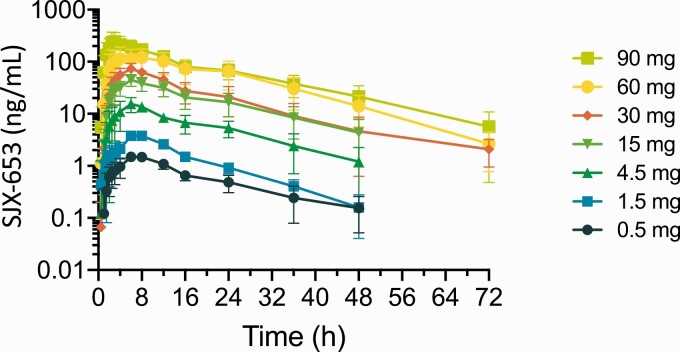
SJX-653 plasma concentration by cohort for doses 0.5–90 mg, over 48 hours, following single oral administration. Data are mean ± SD, n = 4 per cohort. Abbreviation: SD, standard deviation.

Plasma concentrations of SJX-653 for each cohort are shown in Fig. 1. For Cohorts 1 to 6 (0.5–60 mg), median T_max_ values for SJX-653 were consistently at 6.0 hours, whereas for Cohort 7 (90 mg) the median T_max_ was 3.0 hours (Table 3). Mean C_max_ values increased in a dose-proportional manner with an approximate 175-fold increase over the 180-fold increase in dose. Similarly, mean AUC_0-24_ values increased in an approximate dose-proportional manner with a 143-fold increase over the 180-fold increase in dose. Mean t_1/2_ for SJX-653 was similar for all doses and ranged from 9.8 ± 2.4 hours at the 1.5 mg dose, the lowest dose for which t_1/2_ could reliably be determined, to 12.5 ± 3.7 hours at the 15 mg dose, supportive of once-daily (QD) dosing at steady state (Table 3). The PK profile exhibited an approximately 3:1 ratio between C_max_ and plasma concentration at 24 hours, suggesting a low peak trough ratio at steady-state after QD dosing.

**Table 3.** SJX-653 pharmacokinetic parameters

Dose	T_max_ (h)	C_max_ (ng/mL)	AUC_0-24_ (h*ng/mL)	t_1/2_ (h)
0.5 mg	6.5 ± 1.0	1.51 ± 0.3	20.5 ± 4.8	8.88
1.5 mg	6.5 ± 1.0	3.84 ± 0.3	48.8 ± 6.9	9.75 ± 2.4
4.5 mg	6.5 ± 1.0	15.7 ± 4.7	205 ± 74.8	11.3 ± 3.4
15 mg	6.0 ± 0.0	45.0 ± 11.5	615 ± 198	12.5 ± 3.7
30 mg	6.0 ± 0.0	73.0 ± 19.0	919 ± 312	10.3 ± 2.7
60 mg	6.0 ± 1.6	150 ± 84.3	2110 ± 1110	10.8 ± 2.6
90 mg	4.13 ± 2.6	265 ± 106	2940 ± 786	12.2 ± 2.9

Data are mean ± SD. n = 4 for all cohorts except t_1/2_ at 0.5 mg, n = 1 as this parameter was not calculable for other data at this dose.

### Pharmacodynamics

**Figure 2.**
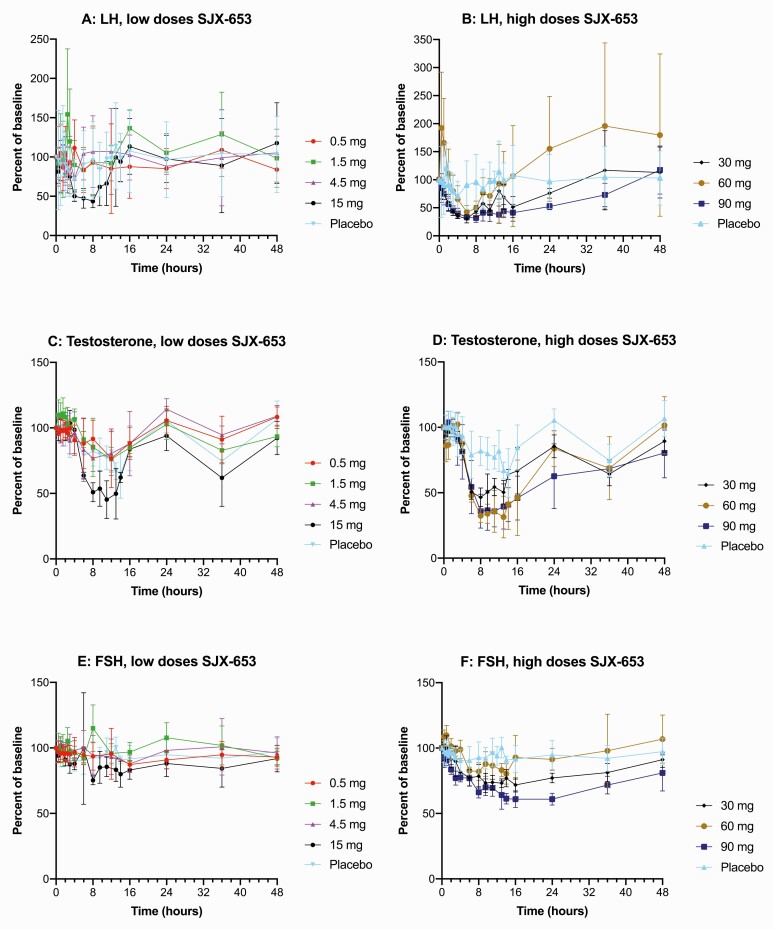
Effects of single doses of SJX-653 on LH (**A**, **B**), testosterone (**C**, **D**), and FSH (**E**, **F**). Doses as indicated on figure. Data are time profiles for changes from baseline calculated as a ratio of mean value for each time point to the mean baseline value, which was calculated as the average of 4 predose time points collected over 1.5 hours before dosing. Mean ± SD, n = 4 subjects per dose; placebo cohorts were pooled, n = 14. Abbreviations: FSH, follicle stimulating hormone; LH, luteinizing hormone; SD, standard deviation.

Single oral doses of SJX-653 resulted in dose-dependent and reversible reductions of plasma concentrations of LH, testosterone, and to a lesser degree FSH (Fig. 2), consistent with previous experience with NK3 antagonists. Table 4 shows LH, FSH, and testosterone concentrations at time 0 and at 6 hours (LH and FSH) and at 8 hours (testosterone) postdose for all groups.

**Table 4.** LH, FSH, and total testosterone concentrations at time 0 and at 6 hours (LH and FSH) and 8 hours (testosterone)

Treatment Group/Dose of SJX-653	n	Time 0 LH (IU/L)	6-hour LH (IU/L)	Time 0 FSH (IU/L)	6-hour FSH (IU/L)	Time 0 Testosterone (nmol/L)	8-hour Testosterone (nmol/L)
Placebo	14	3.8 ± 2.0	3.5 ± 1.4	4.2 ± 2.5	3.8 ± 2.4	17.4 ± 3.9	14.2 ± 2.3
0.5 mg	4	4.2 ± 1.3	3.4 ± 0.4	5.3 ± 0.9	5.3 ± 2.2	15.4 ± 5.7	15.1 ± 6.5
1.5 mg	4	4.6 ± 2.6	3.3 ± 1.8	3.7 ± 2.5	3.4 ± 2.4	15.7 ± 1.6	13.2 ± 2.4
4.5 mg	4	4.7 ± 1.8	5.1 ± 0.9	4.7 ± 1.0	5.0 ± 1.3	15.5 ± 1.9	11.6 ± 2.9
15 mg	4	4.7 ± 1.0	2.1 ± 0.3	4.9 ± 2.6	5.5 ± 5.0	19.0 ± 1.3	9.2 ± 0.4
30 mg	4	4.7 ± 1.3	1.4 ± 0.6	3.7 ± 2.0	2.8 ± 1.4	20.4 ± 3.4	9.2 ± 1.6
60 mg	4	3.4 ± 2.1	1.1 ± 0.4	5.3 ± 4.0	3.1 ± 1.7	13.2 ± 4.8	4.8 ± 2.1
90 mg	4	4.9 ± 1.6	1.7 ± 0.5	4.7 ± 3.0	4.5 ± 3.8	14.1 ± 2.5	5.2 ± 2.8

All data are mean ± SD. Abbreviations: FSH, follicle stimulating hormone; LH, luteinizing hormone.

The majority of time points between 3 and 8 hours showed separation from placebo (*P* < 0.05) for LH reduction at doses of 15 and 30 mg, and between 3 to 24 hours at a dose of 90 mg (Fig. 2A). Reduction of LH at 60 mg relative to placebo was less pronounced compared to 30 and 90 mg, which is likely related to a low LH baseline in the 60 mg cohort resulting in a smaller calculated reduction from baseline compared to other dose cohorts. A significant reduction of testosterone in SJX-653 treated subjects vs placebo was observed at the majority of time points from 6 to 12.5 hours (*P* < 0.05) for doses ≥15 mg, and was significantly different (*P* < 0.05) for the majority of time points up to 24 hours for doses ≥30 mg (Fig. 2B). The duration of testosterone reduction was more prolonged at higher doses. Reduction of FSH was more limited but evident at 30 mg and 60 mg doses, and with the 90 mg dose showing separation from placebo (*P* < 0.05) for most time points between 3 and 36 hours (Fig. 2C).

**Figure 3.**
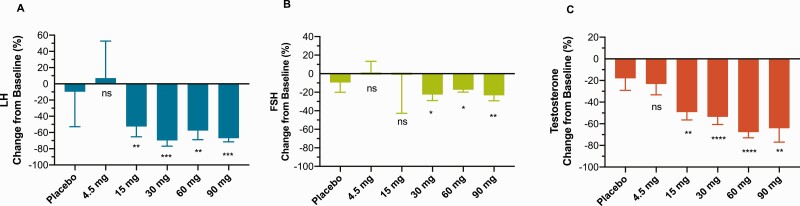
Maximum observed percentage reductions in LH (**A**) and FSH (**B**) at 6 hours and testosterone (**C**) at 8 hours from baseline. Mean ± SD, n = 4 per group except placebo, n = 14. Significance indicated as * *P* < 0.05, ** *P* < 0.01, *** *P* < 0.001, **** *P* < 0.0001 versus placebo. Abbreviations: FSH, follicle stimulating hormone; LH, luteinizing hormone; SD, standard deviation.

At 15 mg, near maximal reductions of LH and testosterone of 56 ± 13% and 55 ± 7%, respectively, were observed after 6 hours for LH and after 8 hours for testosterone, with little to no effect at 4.5 mg, indicating a steep dose-response curve from 4.5 to 15 mg. The maximal mean LH reduction in subjects treated with SJX-653 was 70 ± 7% (mean ± standard deviation) at 6 hours (30 mg) versus 10±43% in placebo-treated subjects at the same time point (*P* < 0.001) (Fig. 3A). The maximal mean testosterone reduction in subjects treated with SJX-653 was 68 ± 5% at 8 hours (60 mg) versus 18 ± 11% in placebo-treated subjects at the same time point (*P* < 0.0001) (Fig. 3B). Testosterone concentration below the normal range (<8.5 nmol/L) occurred postdose in 2/14 subjects in the placebo group and 1, 0, 0, 2, 2, 4, and 3 subjects (each n = 4) in the cohorts treated with 0.5, 1.5, 4.5, 15, 30, 60, and 90 mg SJX-653, respectively, thus in 7/8 men treated with the highest 2 doses, indicating a dose-response relationship.

**Figure 4.**
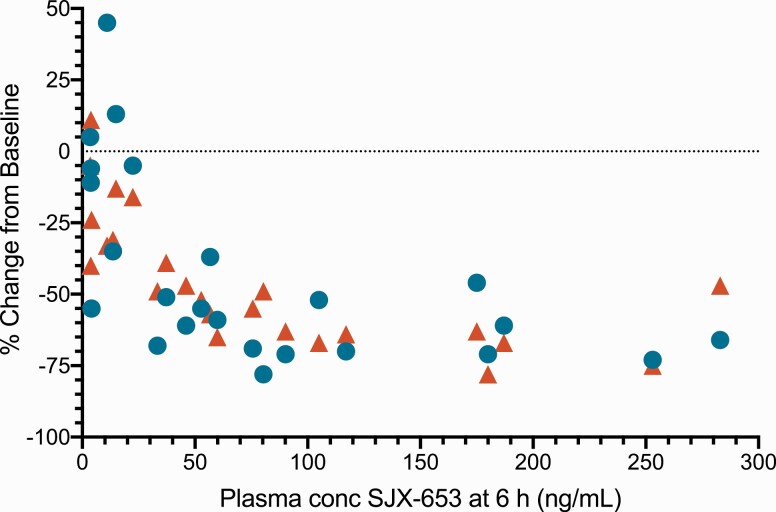
Reduction in LH (6 hours) and testosterone (8 hours) versus plasma concentration of SJX-653 (6 hours): individual subject responses at doses of 1.5 to 90 mg SJX-653. Blue circles: LH at 6 hours; red triangles: testosterone at 8 hours after drug administration. Abbreviation: LH, luteinizing hormone.

The relationship of LH and testosterone reduction from baseline with SJX-653 plasma concentration is shown in Fig. 4. Both markers showed a similar response when accounting for a time lag of 2 hours between changes in LH and changes in testosterone. The maximum reductions from baseline for LH and testosterone were observed at plasma concentrations of approximately 50 ng/mL and higher, with an estimated half maximal inhibitory concentration (IC_50_) of about 30 ng/mL.

## Discussion

This study evaluated the safety, PK, and PD of single oral doses of the novel selective NK3R antagonist SJX-653 in healthy adult men. SJX-653 was safe and well tolerated and resulted in marked, reversible suppression of LH and testosterone levels, with a more limited suppression of FSH. Doses of 0.5–90 mg of SJX-653 showed a dose-proportional increase in exposure with median T_max_ of 6 hours (except for the highest dose). Once-daily (QD) dosing in future studies with repeated drug administration is supported by the t_1/2_ of 10 to 13 hours.

A dose-dependent reduction of LH and testosterone levels achieved near maximal effect after single doses of 15 mg, with an estimated half maximal inhibitory concentration (IC_50_) of about 30 ng/mL, indicating the high potency of SJX-653 as an NK3R antagonist.

This finding provides further evidence for a key role of the NKB-NK3R pathway in the physiological regulation of the male hypothalamo-pituitary-gonadal axis (3) and is in line with data from the administration of another selective NK3R antagonist, MLE4901 (previously called AZD4901), which resulted in reduction of LH, FSH, and testosterone when administered twice daily to healthy men for 7 days (7). Similarly, the NK3R antagonist, ESN364 (fezolinetant), resulted in reversible suppression of LH and testosterone in healthy men (6). Suppression of FSH levels occurred at high doses of SJX-653, albeit to a much smaller extent than inhibition of LH and testosterone. FSH secretion is less dependent on the pulsatile nature of GnRH secretion (20), and findings of changes in FSH secretion have been less consistent than changes in LH in studies investigating the effect of NK3R antagonists in both men and women (6, 7, 10, 11). FSH levels were not significantly suppressed with ESN364 (6). The much smaller reduction in FSH than LH/testosterone secretion observed in this study is thus supportive of the mechanism of T and LH reduction in men via the inhibition of GnRH secretion as a PD marker of NK3R antagonism in men. In addition, the delayed time course of reduction of testosterone versus LH, identified here due to the frequent sampling schedule, supports that the mechanism of action of SJX-653 on testosterone is through the suppression of GnRH and thus LH secretion.

These data provide clear evidence of clinical proof of mechanism of SJX-653 as a potent NK3R antagonist, supporting further development in menopausal VMS and potentially also in sex hormone–dependent conditions. NKB/NK3R signaling in the thermoregulatory neurons of the preoptic area is increasingly understood to have a key role in mediating VMS associated with estrogen deficiency at menopause (14). Elegant opticogenetic labeling experiments have shown that the stimulation of these thermoregulatory neurons by KNDy neurons is mediated by neurokinin B acting though NK3 receptors (15). Three compounds with NK3R antagonist activity have now been shown to have striking efficacy in reducing menopausal VMS (9, 16–18, 21), supporting the development of NK3R antagonists as a highly effective nonhormonal class of medicine. In relation to the possible therapeutic potential in a range of sex hormone–dependent conditions through the inhibition of GnRH secretion, it is noteworthy that the suppression of LH and testosterone was maximal at about 6 to 12 hours, with recovery thereafter, even with higher doses, despite the continuing presence of effective serum concentrations of SJX-653 for at least 48 hours. This indicates the presence of important counter-regulatory pathways to NKB in the control of GnRH secretion. Similarly, the maximal suppression of LH and testosterone achieved was approximately 70%, which is less complete than can be achieved with GnRH antagonists (22). Regulation of GnRH secretion through the KNDy pathway has been likened to a “dimmer switch” (3) in contrast to the effect of GnRH antagonism. The prevention of complete suppression of the reproductive axis may be of therapeutic value in some conditions, as evidenced by the frequent need for “add back” estrogen in the treatment of uterine fibroids and endometriosis using GnRH analogues to reduce otherwise overly troublesome symptoms of estrogen deficiency and to mitigate bone mineral loss (23, 24). Similarly, partial suppression of LH secretion by an NK3 antagonist reduced serum testosterone levels in women with PCOS (11).

In conclusion, this study demonstrates that SJX-653 is a potent NK3R antagonist, as demonstrated by the reduction of established PD markers of NK3R antagonism in healthy men, with approximately 70% suppression of both LH and testosterone after a single oral dose. The plasma half-life of 10 to 13 hours at effective doses supports once daily (QD) administration, and no safety concerns were identified. The striking efficacy that NK3R antagonism has shown that reducing menopausal VMS occurs in the same dose range associated with LH and testosterone reductions in men. Based on the data presented here, SJX-653 demonstrated potent NK3 antagonistic activity, which may be applicable in the treatment of menopausal VMS and in the treatment of sex hormone–dependent clinical conditions.

## Data Availability

The datasets generated during and/or analyzed during the current study are not publicly available but are available from the corresponding author on reasonable request.

## Additional Information

***Disclosure Summary:*** R.A.A. has undertaken consultancy work for Merck, Ferring, Sojournix, Inc., and NeRRe Therapeutics. J.C., D.G., D.T., D.C., P.G., and R.T.-E. are or were employees of Sojournix, Inc. The other authors report no conflicts of interest.
